# Mercury and hydroquinone content of skin toning creams and cosmetic soaps, and the potential risks to the health of Ghanaian women

**DOI:** 10.1186/s40064-016-1967-1

**Published:** 2016-03-11

**Authors:** Eric Selorm Agorku, Edward Ebow Kwaansa-Ansah, Ray Bright Voegborlo, Pamela Amegbletor, Francis Opoku

**Affiliations:** Department of Chemistry, Kwame Nkrumah University of Science and Technology, Kumasi, Ghana

**Keywords:** Total mercury, Hydroquinone, Skin-lightening, Creams, Soaps

## Abstract

In this study, sixty-two (62) skin-lightening creams and soaps were analysed for total mercury and hydroquinone levels. Total mercury was determined by the Cold Vapour Atomic Absorption Spectrophotometry using an automatic mercury analyser and hydroquinone by High Performance Liquid Chromatography. The mean concentration of total mercury in skin toning creams and cosmetic soaps were 0.098 ± 0.082 and 0.152 ± 0.126 μg/g, respectively. The mean concentration of hydroquinone was 0.243 ± 0.385 and 0.035 ± 0.021 % in skin toning creams and cosmetic soaps, respectively. All the creams and soaps analysed had mercury and hydroquinone levels below the US Food and Drug Administration’s acceptable limit of 1 μg/g and 2 %, respectively. The low levels of mercury and hydroquinone in the creams and soaps analysed in this study therefore do not pose any potential risk to consumers who are mostly women in Ghana.

## Background

In most communities across the globe, fairness is branded as beauty, grace and high social status. This perception encourages most women to engage in skin bleaching. Skin-lightening or bleaching has reached epidemic levels in many nations across the world especially in African countries like Ghana, Nigeria, Mali, Kenya and Tanzania (Harada et al. 2001; Adebajo 2002; Mahe et al. 2004; Lewis et al. 2009; Voegborlo et al. 2008). The production of skin-bleaching products is on the rise and in high demand across the world (Perry 2006). Many African women want to keep their skin toned and beautiful by indulging in skin care products that bleach the skin. Most of these bleaching cosmetic products contain different kinds of harmful chemicals such as hydroquinone, mercury, kojic acid and vitamin C, which can affect their health (Amponsah et al. 2014).

Cosmetic preparations containing mercury for bleaching purpose is an old practice (Al-Saleh and Al-Doush 1997). Mercury is a toxic metal but found usefulness in many cosmetic preparations targeted at skin lightening by suppression of melanin production by the skin (Bourgeosis et al. 1986). Cosmetic products containing mercury in the form of inorganic mercury are mainly used by dark skinned people mostly in developing countries (Barr et al. 1973). Mercury is a volatile element and is harmful to the skin when used in an effort to lighten the skin. However, chronic exposure of the body to mercury at very low concentration can cause long-lasting neurological and kidney impairment (Hutson et al. 1999). Mercury in bleaching preparations can be absorbed through the skin and accumulates in body organs giving rise to severe toxicity (Sah 2012). A study conducted on Tanzanian gold miners who use mercury for amalgamation and people not engaged in gold mining activities revealed that the mercury found in their blood and urine was derived from cosmetic soaps and creams containing mercury (Kahatano et al. 1998). Glahder and Appell (1999) also reported high levels of mercury in imported soaps and creams bought in Tanzanian shops. An investigation of Kenyan women with damaged kidney also revealed that they suffered severely from higher incidence of nephritic syndrome, which was attributed to the use of creams containing mercury (Barr et al. 1973).

Hydroquinone is a potential carcinogenic ingredient used in skin lightening and treatment of hyper-pigmentation (Joseph et al. 1998). Hydroquinone does not actually bleach the skin but rather, a strong inhibitor of melanin production (Yoshimura et al. 2001). Hydroquinone used for topical application is known to cause serious health hazards when used excessively (Hutson et al. 1999). Hydroquinone toxicity can lead to severe side effects such as kidney and liver malfunction, blood poisoning, nausea, abdominal pains, convulsion and even coma. Animal test on rats, mice and rabbits showed that hydroquinone can cause acute toxicity (Aldrich 1990).

Most of the skins lightening creams and soaps on the Ghanaian market are imported from USA, Europe, Italy and Cote d’Voire (Voegborlo et al. 2008). The demand for skin-lightening products in Ghana is on the increase despite the health hazards associated with the use of these products. However, data on mercury and hydroquinone levels in skin-lightening soaps and creams sold on the Ghanaian market are lacking and only little work has been done on mercury (Voegborlo et al. 2008) even though concerns have been raised on the negative effect of skin-lightening creams and soaps on the skin. The aim of this work was to analyse the mercury and hydroquinone content in some skin toning creams and cosmetic soaps using automatic mercury analyser coupled with Cold Vapour Atomic Absorption Spectrophotometry (CV-AAS) and High Performance Liquid Chromatography.

## Methods

Different brands of cosmetic soaps and creams were bought from retail shops and markets in Ghana. A total of sixty-two (62) skin-lightening products comprising of fifty-four (54) creams and eight (8) soaps were obtained. Samples were transported to the laboratory. The soaps were cut into smaller sizes with a stainless steel knife and kept in clean labelled glass vials prior to chemical analysis.

The samples were digested for total mercury determination by a modified version of an open flask procedure developed at the National Institute for Minamata Disease (NIMD) Japan (Akagi and Nishimura 1991). A blank and standard solution digest using standard Hg solution were subjected to the same treatment. Determination of mercury in all the digests were carried out by CV-AAS using an Automatic Mercury Analyser, Model HG-5000 (Sanso Seisakusho Co., Ltd, Japan) equipped with mercury lamp operated at a wavelength of 253.7 nm.

Hydroquinone in all the samples were extracted with ethanol. About 0.10 g of each cream and soap was weighed into a 10 ml flask and 8.0 ml of methanol was added and heated at 40 °C in a water bath and shaken occasionally until it dissolved. The mixture was allowed to cool and made up to the mark with methanol. The solution was filtered using a sintered glass filter with a vacuum pump. Blank and standard hydroquinone solutions were subjected to the same treatment. Determination of hydroquinone was carried out by a chromatographic method using a High Performance Liquid Chromatography, Model Varian 325 (England) equipped with UV/visible and fluorescence detector.

### Method validation

Replicate blanks, recovery tests and reference materials (DORM-2) from the National Research Council (NRC) of Canada were used for the method validation and quality control. The validity of the method has been proved by the agreement between the measured (4.64–4.81 µg/g) and certified (4.21–4.83 µg/g) concentrations in DORM-2 Certified Reference Material. Recovery studies were performed by adding increasing amounts of mercury and hydroquinone standard solutions to three different samples, which were separately taken through similar digestion and extraction procedures respectively. Accepted values range from 95 to 110 % (Miller and Miller 1988). Analytical and matrix recovery studies for mercury ranged from 97 to 102 % with coefficient of variation between 4 and 9 %. The recovery studies of hydroquinone yielded results between 98 and 103 %. The coefficients of variation of hydroquinone concentration in triplicates were between 0.19 and 4.73 %. This shows that the method is accurate and fit for analysis of the above parameters.

## Results and discussion

In all the samples analysed, T-mercury levels ranged from <0.001 to 0.327 ± 0.062 μg/g and hydroquinone concentration ranged from below detection to 1.61 ± 0.72 %. The results for T-mercury and hydroquinone concentrations in skin toning creams and cosmetic soaps are shown in Tables 1 and 2.

**Table 1:** Levels of mercury and hydroquinone concentrations in skin toning creams

Product name	Acronym for product	Mercury (µg/g)	Hydroquinone (%)	Country of origin
Akagni^®^ Skin Toning Cream	AKS	0.129 ± 0.057	0.21 ± 0.16	Cote d’Voire
Body White	BW	<0.001	ND	Cote d’Voire
Carotone	CT	0.147 ± 0.058	0.02 ± 0.01	Cote d’Voire
Cherie Claire	CC	0.008 ± 0.001	0.01 ± 0.00	Cote d’Voire
Claire Plus	CP	0.145±	ND	Cote d’Voire
Clair-Liss Genial	CLG	<0.001	0.04 ± 0.02	Cote d’Voire
Cocoderm	CD	0.067 ± 0.046	0.83 ± 0.51	Cote d’Voire
Cocoon Cocoa Butter	CCB	0.059 ± 0.012	ND	Cote d’Voire
Eclat^®^ Gel	EG	0.327 ± 0.062	ND	Cote d’Voire
Extra Toning Cocoa Butter	ETCC	0.096 ± 0.029	0.04 ± 0.00	Cote d’Voire
Fair and Light	FL	<0.001	ND	Cote d’Voire
G & G	GG	<0.001	0.01 ± 0.00	Cote d’Voire
Méticée	MT	<0.001	0.03 ± 0.01	Cote d’Voire
Miss Caroline	MC	0.256 ± 0.045	0.02±	Cote d’Voire
Palmas Cocoa Butter	PCB	0.001 ± 0.000	ND	Cote d’Voire
PỐlisse	PL	<0.001	ND	Cote d’Voire
Sivoclair	SC	<0.001	ND	Cote d’Voire
Sure White	SW	0.140 ± 0.045	0.01 ± 0.00	Cote d’Voire
Victory white	VW	0.003 ± 0.001	ND	Cote d’Voire
Clear N Smooth Cream	CNS	<0.001	ND	Italy
Esapharma MOVATE Cream	EMC	0.166 ± 0.123	ND	Italy
Fashion Fair Cream	FFC	0.001 ± 0.000	ND	Italy
L’abidjanaise^®^ Cream	LAC	<0.001	ND	Italy
Movate Cream	MTC	0.026 ± 0.003	0.18 ± 0.05	Italy
Naturel Cream	NC	0.008 ± 0.005	ND	Italy
Neo-vate White Cream Plus	NWCP	<0.001	ND	Italy
Neutrotone^®^ Cream	NTC	0.004 ± 0.001	ND	Italy
New Age Cream	NAC	0.250 ± 0.031	ND	Italy
New Skin Light^®^ Cream	NSLC	0.002 ± 0.001	ND	Italy
Perfect Skin	PS	0.122 ± 0.057	ND	Italy
Polux Gel	PG	0.001 ± 0.000	ND	Italy
Skin Maxitoner	SM	0.015 ± 0.004	ND	Italy
Skin Solution Cream	SSC	0.067 ± 0.025	0.51 ± 0.18	Italy
Skinicles Cream	SCC	0.152 ± 0.079	0.19 ± 0.04	Italy
Swiss Formula Cream	SFC	0.046 ± 0.029	0.02 ± 0.01	Italy
Crusader^®^ Ultra Brand	CUB	0.135 ± 0.085	ND	UK
Dermatological E45 Solution	DES	0.192 ± 0.097	ND	UK
Zarina Cream	ZC	0.070 ± 0.031	1.61 ± 0.72	UK
Sukisa Bango	SB	0.228 ± 0.023	ND	UK
Amiderm Cream	AC	0.043 ± 0.015	0.20 ± 0.11	India
Betasol Cream	BC	0.081 ± 0.031	0.20 ± 0.02	India
Chocho Skin Toner	CST	<0.001	ND	India
Closol Cream	CLC	0.060 ± 0.039	0.32 ± 0.16	India
Epiderm™ Cream	EC	<0.001	ND	India
Funbact-A	FA	0.081 ± 0.048	ND	India
Surfaz Cream	SFC	0.182 ± 0.057	0.01 ± 00	India
Dawmy	DW	<0.001	ND	USA
Dr. Fred Summit Skin Whitener	DFSW	0.106 ± 0.028	ND	USA
BioClaire	BCL	<0.001	ND	Indonesia
Hyprogel	HG	0.076 ± 0.015	ND	Germany
So sexy	SS	0.145 ± 0.091	ND	Senegal
Mercy Ointment	MO	<0.001	ND	Ghana
The Karibu D’or Sana	TKD	0.166 ± 0.026	ND	China
Dove Silk Cream	DSC	0.015 ± 0.002	0.40 ± 0.71	Nigeria

*ND* not detected

**Table 2:** Levels of mercury and hydroquinone concentrations in cosmetic soaps

Product name	Acronym for product	Mercury (µg/G)	Hydroquinone (%)	Country of origin
Carotone soap	CTS	0.148 ± 0.085	0.02 ± 0.01	Cote d’Voire
Black Velvet soap	BVS	0.003 ± 0.000	ND	Italy
Classic White soap	CWS	0.204 ± 0.065	0.05 ± 0.03	Indonesia
Pharmapur (soap)	PPS	0.027 ± 0.015	ND	Turkey
Be White Whiteniser soap	BWS	0.214 ± 0.059	ND	UK
Crusader^®^ medicated soap	CMS	0.337 ± 0.073	ND	UK
Skin success complexion toning soap	SCTS	0.017 ± 0.009	ND	UK
Tura soap	TS	0.272 ± 0.089	ND	Nigeria

*ND* not detected

Majority of the skin-lightening creams analysed in this study originated for Cote d’Voire (34 %), Italy (30 %) and India (13 %). The rest originated from UK, USA, Senegal, China, Germany, Nigeria and Ghana. T-mercury concentration (μg/g) in the skin-lightening creams imported from Cote d’Voire ranged from <0.001 to 0.327 ± 0.062, <0.001 to 0.250 ± 0.031 in creams imported for Italy and <0.001 to 0.182 ± 0.057 in creams imported from India. About 28% of the creams recorded T-mercury concentration below detection (<0.001 μg/g). The highest concentration of T-mercury was recorded in EG which is imported from Cote d’Voire. T-mercury concentration in soaps ranged from 0.003 ± 0.000 to 0.337 ± 0.073 μg/g. All the skin-lightening creams and soaps analysed had T-mercury concentration below 1.0 μg/g maximum limit set by the United States Food and Drug Administration (USFDA) (USFDA 2009). Similar results were reported by Voegborlo et al. (2008). However, previous studies conducted by Al-Saleh and Al-Doush (1997) on mercury concentration in creams obtained from the Saudi Arabians market which originated from Asia and Middle East contained T-mercury concentration above the US FDA limit. Results of T-mercury determined in cosmetic products obtained on Dares Salaam market in Tanzania also showed very high concentration of mercury in some creams and soaps above the US FDA limit with T-mercury ranged from 0.11 to 8665 μg/g in cosmetic soaps and 0.16–25.30 μg/g in cosmetic creams (Kinabo 2003).

Hydroquinone levels in the skin-lightening products ranged from below detection to 0.83 ± 0.51 % in creams imported from Cote d’Voire, 0.02 ± 0.01 to 0.51 ± 0.18 % in creams imported from Italy, below detection to 0.32 ± 0.16 % in creams imported from India. Creams originating from USA, Indonesia, Germany, Senegal, Ghana and China recorded hydroquinone levels below the detection limit. The highest concentration of hydroquinone was recorded in ZC (1.61 ± 0.72 %) imported from UK followed by CD (0.83 ± 0.51 %) imported from Cote d’Voire, Skin Solution Cream (0.51 ± 0.18 %) imported from Italy, DSC (0.40 ± 0.71 %) imported from Nigeria and CLC (0.32 ± 0.16 %) imported from India. Hydroquinone levels in the skin-lightening soaps ranged from below detection to 0.05 ± 0.03 %. Classic white soap and CTS recorded hydroquinone concentration of 0.05 ± 0.03 and 0.02 ± 0.01 %, respectively. Overall, 64 % of the creams and 75 % of soaps analysed recorded hydroquinone concentrations below detection. All the skin-lightening creams and soaps analysed recorded hydroquinone concentration below the US FDA threshold limit of 2 % (USFDA 2009). Doreen (2010) recorded levels of hydroquinone (0.001–3.45 %) in some skin lighteners in Ghana, which was higher than the present study. However, levels of hydroquinone (0.0002–0.0350 %) in body creams and lotions by Terer et al. (2013), Kenya are comparable with the present study.

## Conclusion

The results for total mercury and hydroquinone levels in skin lightening creams and soaps obtained in this study are below the limits set by United States Food and Drug Administration (USFDA). The use of these creams and soaps do not pose any health risk to Ghanaian women so far as mercury and hydroquinone levels are concern. Though the samples analysed in this study do not contain high levels of total mercury and hydroquinone, their continuous use may pose health threat since hydroquinone and mercury can accumulate in the liver and kidneys which can cause damage to these organs.

## References

[CR1] Adebajo S (2002). An epidemiological survey of the use of cosmetic skin lightening cosmetics among traders in Lagos, Nigeria. West Afr J Med.

[CR2] Akagi H, Nishimura H, Suzuki T, Imura N, Clarkson TW (1991). Speciation of mercury in the environment. Advances in mercury toxicology.

[CR3] Aldrich (1990). Catalog/Handbook of fine chemicals.

[CR4] Al-Saleh I, Al-Doush I (1997). Mercury content in skin lightening creams and potential hazards to the health of Saudi women. J Toxicol Environ Health.

[CR5] Amponsah D, Sebiawu GE, Voegborlo R (2014). Determination of amount of mercury in some selected skin-lightening creams sold in the Ghanaian market. Int J Eng Res Technol.

[CR6] Barr RD, Woodger BM, Rees PH (1973). Levels of mercury in urine correlated with the use of skin lightening creams. Am J Clin Pathol.

[CR7] Bourgeosis M, Dooms-Goossens A, Knockaert D, Sprenger D, Vsan Boven M, Van tittelboom T (1986). Mercury intoxication after topical application of a metallic mercury ointment. Dermatologica.

[CR8] Doreen A (2010) Levels of hydroquinone and mercury in skin lightening creams and their potential risk to the health of consumers in Ghana. Unpublished Master’s dissertation, Kwame Nkrumah University of Science and Technology, Kumasi, Ghana

[CR9] Glahder CM, Appell PWU (1999) Mercury in soap in Tanzania. NERI Technical Report No. 306. Department of Arctic Environment Geological Survey of Denmark and Greenland, p 19

[CR10] Harada M, Nakachi S, Tasaka K, Sakashita S, Muta K, Yanagida K, Ohno H (2001). Wide use of skin-lightening soap may cause mercury poisoning in Kenya. Sci Total Environ.

[CR11] Hutson DH, Dean BJ, Brooks TM, Hudson-Walker G (1999). Genetic toxicology testing of 41 industrial chemicals. Research.

[CR12] Joseph P, Klein-Szanto AJP, Jaiswal AK (1998). Hydroquinones cause specific mutations and lead to cellular transformator and in vivo tumorgenesis. Br J Cancer.

[CR13] Kahatano JMJ, Mnali SR, Akagi H (1998). A study of mercury levels in fish and humans in Mwakkitolyo mine and Mwanza town in the Lake Victoria gold-fields, Tanzania. Br Med J.

[CR14] Kinabo CP (2003). Comparative analysis of mercury content in human hair and cosmetics products used in Dar es salaam, Tanzania. J Pharm Pharmacol.

[CR15] Lewis K, Robkin N, Gaska K, Njoki LC, Andrews E, Jetha K (2009). The Tanzanian response to dangerous skin bleaching products and practices and the gendered politics of it all: a critical analysis. J Cult Afr Women Stud.

[CR16] Mahe A, Ly F, Gounongbe A (2004). The cosmetic use of bleaching products in Dakar, Senegal: socio-economic factors and claimed motivations. Sciences Sociales Et Sante.

[CR17] Miller JC, Miller JN (1988). Statistics for analytical chemistry.

[CR18] Perry I (2006). Buying white beauty. Cardozo J Law Gend.

[CR19] Sah RC (2012). Poisonous cosmetics, the problem of mercury in skin whitening creams in Nepal, vi+10.

[CR20] Terer EK, Magut H, Mule S (2013). UV–Vis analysis and determination of hydroquinone in body lotions and creams sold in retail outlets in Baraton, Kenya. Baraton Interdispl Res J.

[CR21] USFDA (2009) United State Food and Drug Administration supporting information for toxicological evaluation by the National toxicology program 21 May 2009

[CR22] Voegborlo RB, Agorku ES, Buabeng-Acheampong B, Zogli E (2008). Total mercury content of skin toning creams and the potential risk to the health of women in Ghana. J Sci Technol.

[CR24] Yoshimura K, Tsukamoto K, Okazaki M, Virador VM, Lei T-C, Suzuki Y, Uchida G, Kitano Y, Harii K (2001). Effects of all-trans retinoic acid on melanogenesis in pigmented skin equivalents and monolayer culture of melanocytes. J Dermatol Sci.

